# Contribution of parvalbumin and somatostatin-expressing GABAergic neurons to slow oscillations and the balance in beta-gamma oscillations across cortical layers

**DOI:** 10.3389/fncir.2015.00006

**Published:** 2015-02-03

**Authors:** Toshinobu Kuki, Kazuyuki Fujihara, Hideki Miwa, Nobuaki Tamamaki, Yuchio Yanagawa, Hajime Mushiake

**Affiliations:** ^1^Department of Physiology, Graduate School of Medicine, Tohoku UniversitySendai, Japan; ^2^Department of Genetic and Behavioral Neuroscience, Gunma University Graduate School of MedicineMaebashi, Japan; ^3^Department of Psychiatry and Human Behavior, Gunma University Graduate School of MedicineMaebashi, Japan; ^4^Core Research for Evolutional Science and TechnologyTokyo, Japan; ^5^Department of Morphological Neural Science, Graduate School of Medical Sciences, Kumamoto UniversityKumamoto, Japan

**Keywords:** PV cells, slow oscillation, gamma oscillation, beta oscillation, neocortex, CSD, wavelet analysis, mouse

## Abstract

Cortical interneurons are classified into several subtypes that contribute to cortical oscillatory activity. Parvalbumin (PV)-expressing cells, a type of inhibitory interneuron, are involved in the gamma oscillations of local field potentials (LFPs). Under ketamine-xylazine anesthesia or sleep, mammalian cortical circuits exhibit slow oscillations in which the active-up state and silent-down state alternate at ~1 Hz. The up state is composed of various high-frequency oscillations, including gamma oscillations. However, it is unclear how PV cells and somatostatin (SOM) cells contribute to the slow oscillations and the high-frequency oscillations nested in the up state. To address these questions, we used mice lacking glutamate decarboxylase 67, primarily in PV cells (PV-GAD67 mice) or in SOM cells (SOM-GAD67 mice). We then compared LFPs between PV-GAD67 mice and SOM-GAD67 mice. PV cells target the proximal regions of pyramidal cells, whereas SOM cells are dendrite-preferring interneurons. We found that the up state was shortened in duration in the PV-GAD67 mice, but tended to be longer in SOM-GAD67 mice. Firing rate tended to increase in PV-GAD67 mice, but tended to decrease in SOM-GAD67 mice. We also found that delta oscillations tended to increase in SOM-GAD67 mice, but tended to decrease in PV-GAD67 mice. Current source density and wavelet analyses were performed to determine the depth profiles of various high-frequency oscillations. High gamma and ripple (60–200 Hz) power decreased in the neocortical upper layers specifically in PV-GAD67 mice, but not in SOM-GAD67. In addition, beta power (15–30 Hz) increased in the deep layers, specifically in PV-GAD67 mice. These results suggest that PV cells play important roles in persistence of the up state and in the balance between gamma and beta bands across cortical layers, whereas SOM and PV cells may make an asymmetric contribution to regulate up-state and delta oscillations.

## Introduction

Parvalbumin (PV)-expressing cells are the major type of inhibitory gamma-aminobutyric acid-producing (GABAergic) interneurons of the neocortex. These cells are distributed across cortical layers, target the proximal regions of pyramidal cells, and are characterized by a fast-spiking physiological phenotype (Kawaguchi, 1993; Kawaguchi and Kubota, 1993; Condé et al., 1994; Tamamaki et al., 2003). In contrast, somatostatin (SOM)-expressing cells are dendrite-preferring interneurons (Martinotti, 1889; Fairèn et al., 1984; Somogyi and Cowey, 1984). PV cells are involved in generating gamma (γ) oscillations in local field potentials (LFPs) (Freund, 2003; Fuchs et al., 2007; Sohal et al., 2009; Buzsáki and Wang, 2012). Gamma oscillations are found in the upper layers in particular (Roopun et al., 2006).

During ketamine-xylazine anesthesia or sleep, slow oscillations occur in the cortex (Steriade et al., 1993a,b,c; Destexhe et al., 1999; Timofeev et al., 2000). A slow oscillation is an alternation between the active up state and silent down state at ~1 Hz (Wilson and Kawaguchi, 1996; Anderson et al., 2000; Kitano et al., 2002), and also triggers and groups delta oscillations (1–4 Hz; Amzica and Steriade, 1998). Various high-frequency oscillations, including γ oscillations, are nested within the up state (Steriade et al., 1996; Hasenstaub et al., 2005; Ruiz-Mejias et al., 2011). The up state is an intriguing model of high-frequency activity, which is a self-organized ensemble of neurons maintaining balanced excitatory and inhibitory inputs.

Many studies have investigated the up state mechanism. The up state is generated by deep pyramidal neurons and interneurons, including PV cells. Deep-layer 5 pyramidal neurons fire earlier than neurons in other layers during the up state (Sanchez-Vives and McCormick, 2000). Layer-specific inhibition studies show that deep layer pyramidal neurons are necessary during the up state (Wester and Contreras, 2012; Beltramo et al., 2013). Fast-spiking interneurons may initiate the up state via thalamo-cortical inputs (Puig et al., 2008; Ushimaru et al., 2012). Pacemaker-like Martinotti cells in layer 5 may also initiate the up state (Le Bon-Jego and Yuste, 2007). Furthermore, a GABA(A) receptor antagonist shortens up state duration, suggesting that GABAergic interneurons help maintain the up state (Mann et al., 2009; Sanchez-Vives et al., 2010). In addition, a study of patients with schizophrenia demonstrated that GABA-synthesizing enzyme glutamate decarboxylase 67 (GAD67) content is lower in PV cells, and that their network activity (i.e., γ oscillations) is impaired, suggesting a link between GAD67 in PV cells and γ oscillations (Lisman et al., 2008). GABAergic circuits including PV cells also develop multisensory integration in the insular cortex, a deficiency of which may be relevant to schizophrenia and autism (Gogolla et al., 2014).

However, it remains unclear how interneuronal subtypes contribute to the persistence of the up state and high-frequency oscillations within the up state. Therefore, we propose two hypotheses. The first is that PV cells contribute to maintain the up state. The second is that PV cells are also involved in balancing the high-frequency oscillations across cortical layers. To test these hypotheses, we examined the up state of slow oscillations and high frequencies using mice lacking GAD67 in PV or SOM cells. We recorded LFPs along the cortical layers of knockout mice under ketamine and xylazine anesthesia. We also applied wavelet transformation to the CSD data (Lakatos et al., 2005; Maier et al., 2011).

## Materials and methods

### Animals

All animal experiments were approved by the Tohoku University Committee for Animal Research, The Animal Care and Experimentation Committee of Gunma University, Showa Campus, and The Animal Research Committee of Kumamoto University. Every effort was made to minimize the number of animals used and their suffering. To achieve a homozygous deletion of the GAD67 gene primarily in PV cells or in SOM cells, GAD67-floxed mice (Obata et al., 2008) were crossed either with PV-Cre mice expressing Cre recombinase (Cre) under control of a bacterial artificial chromosome transgenic PV promoter fragment (Tanahira et al., 2009) or with SOM-IRES-Cre mice expressing Cre under control of an endogenous SOM promoter (Taniguchi et al., 2011). We refer to the GAD67^flox/flox^/PV-Cre mice, the GAD67^flox/flox^/SOM^IRES−Cre/+^ mice, and the GAD67^flox/flox^ mice as PV-GAD67 mice, SOM-GAD67 mice, and control mice, respectively, hereafter. The mice were of either sex. The PV-GAD67 mice and control mice were littermates, and the SOM-GAD67 mice and their control mice were also littermates.

### Surgical procedures

Surgical anesthesia was induced by intraperitoneal injection of ketamine hydrochloride (100 mg/kg) and xylazine (10 mg/kg). All surgical or pressure points were treated with 0.25% lidocaine hydrochloride. The anesthesia plane for the surgery was determined by the absence of a vibrissa motor response, a leg withdrawal response, and an eye-blink reflex. Additional doses of the ketamine and xylazine mixture were injected intraperitoneally, as required. After placing the animal on a stereotaxic apparatus, a small craniotomy and duratomy were made above the somatosensory area (1.5 mm lateral and 1.6 mm rostral from bregma) on the right hemisphere to insert the recording electrode. The coordinates were determined according to Franklin and Paxinos (2008). A skull screw was placed above the cerebellar cortex of the left hemisphere as a voltage reference.

### Electrophysiological recordings

Electrophysiological recordings were taken using single-shank electrodes with a linear array of 16 recording channels (16 channels, 100-μm inter channel spacing; A1 × 16–10 mm, 100–413; Neuronexus, Inc., Ann Arbor, MI, USA). The distance between the two channels was 100 μm. The impedance of the probes was ~0.7 mW. The signals were amplified at unity gain using a head stage amplifier (HST/8o50-G1-GR; Plexon, Inc., Dallas, TX, USA) and were further amplified (gain: 250×) and recorded using a 16-channel recording system (Omniplex; Plexon) at a 40-kHz sampling rate. Then, the signal was split into LFP (~200 Hz) and multi-unit activity (MUA; ~300 Hz) ranges by analog filtering. LFP was down-sampled at 1000 Hz. We recorded the signal in each animal for about 30 min. Stable representative data used for all analyses were defined as those between anaesthesias for about 1 min in each animal (about 30 up states in each animal). Total data used for analysis was about 20 min.

### Perfusion and histology

After the recording session, we electrified the recording sites using a multi-channel probe to mark probe position. Next, 5 μA of electricity was passed until the sum of electricity reached 400 μC. The mice were then euthanized with intraperitoneal pentobarbital (120 mg/kg). After voluntary breathing stopped, the mice were transcardially perfused with phosphate buffer (PB; 0.4 M, pH 7.4), followed by 100 mL 4% formaldehyde (FA) in PB. The whole brain was removed and post-fixed in 4% FA overnight at 4°C. After cryoprotection using a graded series of sucrose solutions (10, 20, and 30% in PB) at 4°C, each brain specimen was frozen using compressed CO_2_ and sliced at 40 μm using a rotary microtome (TU-213; Yamato Kohki Industrial, Saitama, Japan). The slices were mounted on gel-coated slides (MAS-GPtypeA S9901 white; Matsunami Glass Ind., Ltd., Osaka, Japan), dried, and stained using cresyl echt violet and coverslipped. The stained slides were inspected and photographed microscopically (BZ-9000; Keyence, Osaka, Japan) to verify the recording loci.

### Data analysis

Wavelet analyses were performed using a custom-written program in R (using function cwt() in package “Rwave”). Briefly, the LFP recording, *f*(*t*), was transformed as follows (Mizuhara and Yamaguchi, 2011):

(1) $$W\left(b,\;a\right)\;=\;\frac{1}{\sqrt{a}}\int_{\text{–}\infty }^{\infty }f(t)G(\frac{t\text{–}b}{a}),$$

where *a*, *b* denote the scaling factor (1/Hz) and the center location (ms) of the mother wavelet function, respectively. 1/*a* varied from 0.1 to 500 Hz.

In Equation (1), *G*(*x*) is the complex Gabor function:

(2) $$G\left(x\right)\;=\;\frac{1}{\sqrt{\text{2}\pi }}\;exp(-{\left(\frac{x}{2Fs}\right)}^{2})\text{exp}(\frac{2\pi i}{Fs}x),$$

where *Fs* is the sampling rate (1000/s).

In **Figures 2**, **5**, the wavelet power spectrum (WPS), |*W* (*b*, *a*)|, from each LFP recording is shown. The WPS was scaled linearly from zero to one and color-coded. In **Figures 5**, **6**, the WPS was sorted according to each high frequency band: alpha (7–14 Hz), beta (15–30 Hz), low gamma (30–60 Hz), high gamma (60–90 Hz), and ripple (100–200 Hz) bands. To detect the up state, the mean of all channel WPS across high frequency bands (10–100 Hz) was used (semi-transparent red color in **Figures 2**, **4**, **5**). To reduce any fluctuations unrelated to the up state, we applied simple moving mean to WPS:

(3) $$M\left(t\right)\;=\;\sum_{T\;=\;t-\frac{n}{2}}^{t\;+\;\frac{n}{2}}WPS\left(T\right)/(n\;*\;Fs\;+\;1),$$

where *n* is the width of the simple moving mean (0.25 s), *Fs* is the sampling rate (1000 /s), and *t* is the central time. We set the state criteria as half the power of the largest *M*(*t*) (mean of top 5%). We determined those *M*(*t*) with a spectral power larger than the criteria to be up state and the others as down state. The mean trigger of the up state was calculated according to the 400 ms before and after the up state (**Figures 4B,D,F**, **6B,D,F**). All data from each animal were averaged at the start of the up state for the statistical analyses.

For CSD analysis, each of the LFP recordings was filtered across channels using Hanning windowing to remove high-frequency noise (Rappelsberger et al., 1981):

(4) $$\bar{f_{n}}(t)=0.23f_{n-1}(t)+\;0.54f_{n}(t)+\;0.23f_{n+1}(t),$$

where *f*_*n*_(*t*) is the field potential recorded at the *n*th channel.

The following formula was used to obtain the (relative) CSD (Nicholson and Freeman, 1975; Mitzdorf and Singer, 1977; Rappelsberger et al., 1981; Chauvette et al., 2010):

(5) $${\bar{I}}_{n}\left(t\right)\;=\;-({\bar{f}}_{n-1}\left(t\right)-2{\bar{f}}_{n}\left(t\right)\;+\;{\bar{f}}_{n+1}\left(t\right)).$$

In this formula, the resistance of the neural tissue was assumed to be constant across the cortical layer, and the other physical constants were ignored for simplicity. Relative WPS of CSD was calculated by measuring the ratio of the WPS within the band of interest to the total power of the high frequency bands (from alpha to ripple) within each channel (**Figures 5**, **6**; Carlén et al., 2011).

### Statistical analysis

*N* always refers to the number of animals. Welch's two-sample *t*-tests were used together with the Benjamini–Hochberg correction. The normalized values are shown in **Figure 3**, separated by values for the control mice to increase clarity. The differences in **Figure 6** were calculated by subtracting the values for knockout mice from those for the control mice. All statistical tests are based on data without subtraction or normalization.

## Results

### Depth profile of slow oscillations across the cortical layers

**Figure 1:**
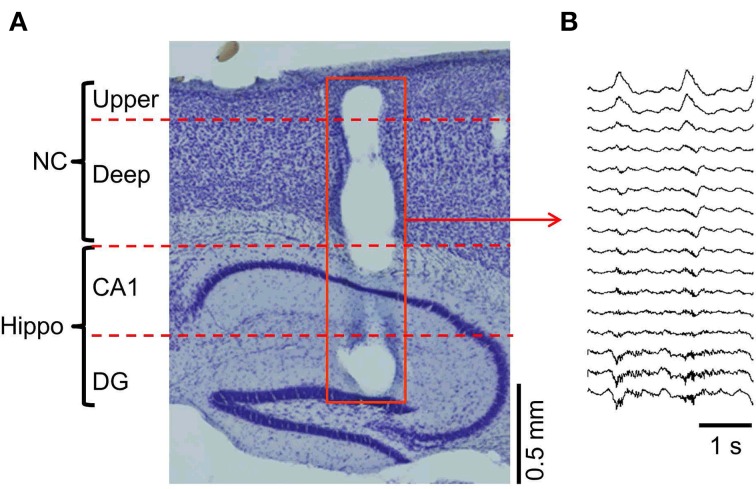
**Multi-channel probe positions and LFP recordings. (A)** The multi-channel probe was positioned across the cortico-hippocampal area (NC, neocortex; upper, upper layers; deep, deep layers; CA1, hippocampal area around the Cornu Ammonis areas; DG, hippocampal area around the dentate gyrus). The hole on the sagittal section (red square) shows the position of the probe. **(B)** The temporal profile example of the LFP recorded from the probe in **(A)**.

We recorded LFPs across the cortical layers during slow oscillations under ketamine anesthesia using a 16-channel silicon probe (Figures 1A,B). Our region of interest was the neocortex, although the probe tip reached the hippocampus underneath the neocortex. The slow oscillations were characterized by synchronous oscillation (<1 Hz) throughout the cortico-hippocampus, whereas polarity reverses with depth of the cortical layer (Steriade et al., 1993a,b; Chauvette et al., 2010). In addition, the high-frequency power was stronger in the area around the hippocampus than that near the neocortex (Wolansky et al., 2006; Sharma et al., 2010).

### Selective decrement of the up state duration in PV-GAD67 mice

**Figure 2:**
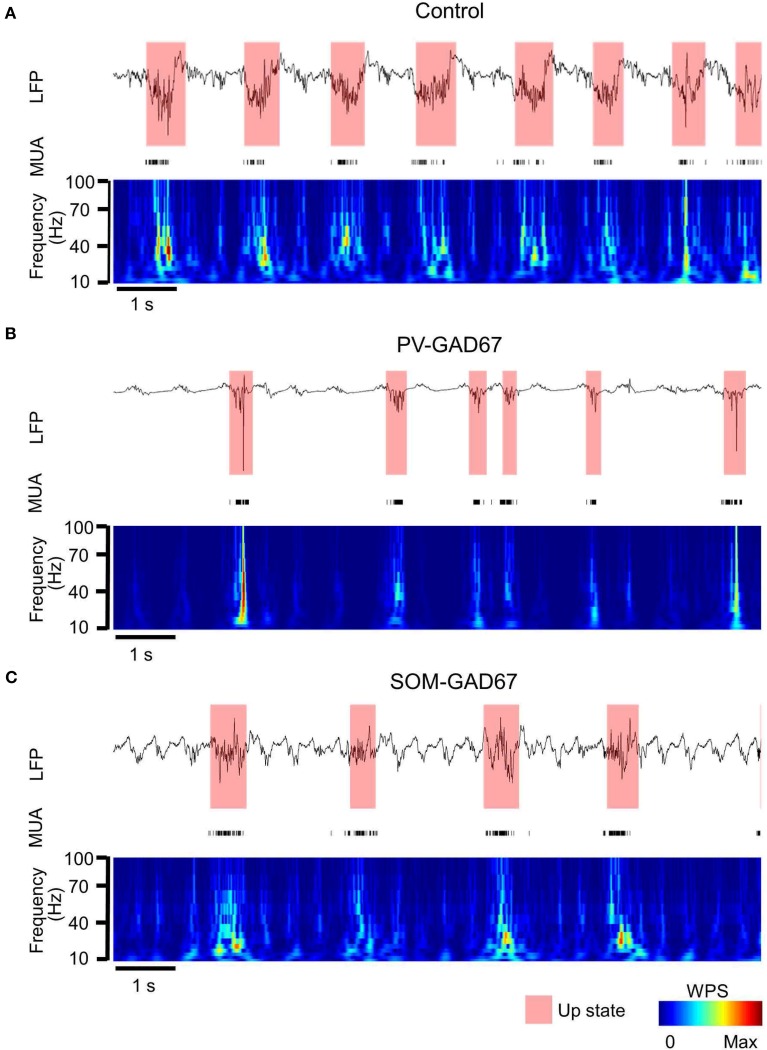
**LFP and MUA temporal profile and the up state detected from the WPS of the LFP. (A)** The LFP temporal profile example from channel 14 (top), the MUA temporal profile from all channels (middle), and the WPS (bottom) of a control mouse (semi-transparent red, up state detected from the WPS; colored bar, normalized amplitude of WPS). **(B)** PV-GAD67 mouse under the same conditions described in **(A)**. The up state tended to be shorter and the down state longer in duration compared with the control mouse. **(C)** SOM-GAD67 mouse under the same conditions described in **(A)**. The down state tended to be longer in duration compared with that in the control mouse.

**Figure 3:**
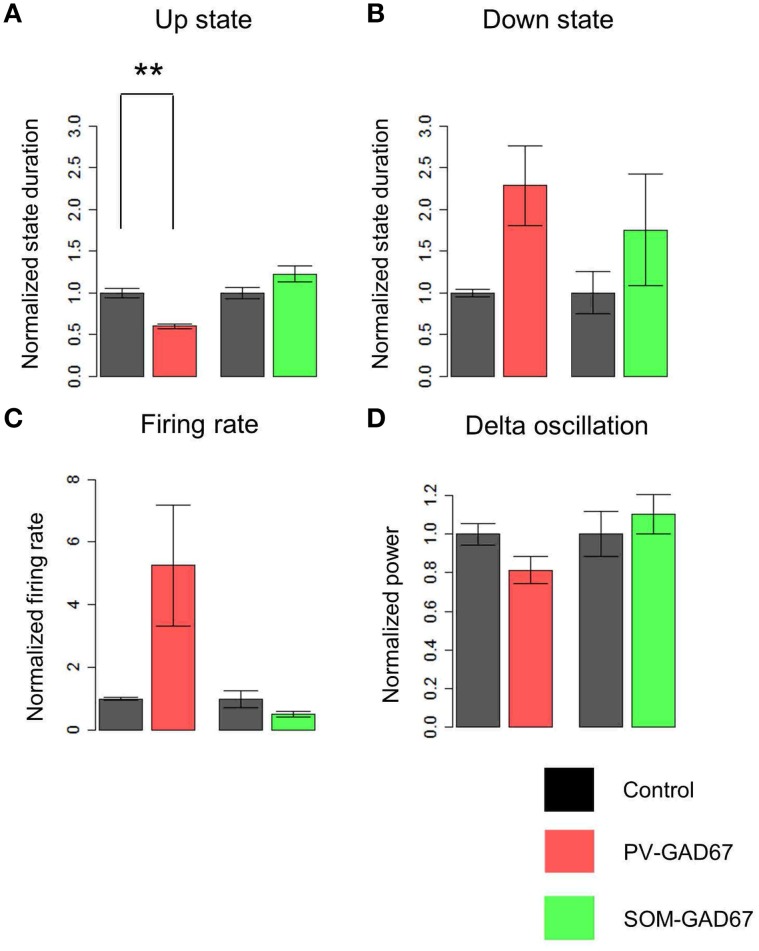
**A comparison of the state duration, delta oscillation, and firing rate of MUA of PV-GAD67 and SOM-GAD67 with control mice**. **(A)** A comparison of the normalized duration average of the up state between PV-GAD67 or SOM-GAD67 mice and control mice (red bar, the normalized duration of the up state of PV-GAD67 mice; green bar, the normalized duration of the up state of SOM-GAD67 mice; black bar, the normalized duration of the control mice). **(B)** A comparison of the normalized down state average under the same conditions described in **(A)**. **(C)** A comparison of the normalized firing rate average of MUA under the same conditions described in **(A)**. **(D)** A comparison of the normalized delta oscillation power average under the same conditions described in **(A)**. Welch's two-sample *t*-test together with Benjamini-Hochberg correction, *N* = 5 in PV-GAD67 mice, *N* = 4 in SOM-GAD67 mice, *N* = 9 in control mice; ^**^P < 0.01, error bars indicate SEM.

We investigated the role of PV-expressing cells (PV cells) in cortical oscillatory activity using PV-GAD67 mice and SOM-GAD67 mice in which GAD67 was ablated primarily in PV or SOM cells, respectively. We first assessed the effects of the functional deficiency on slow oscillations between PV and SOM cells, and then compared the state durations and firing rates of PV-GAD67 and SOM-GAD67 mice with those of control mice. We detected up and down states by quantitatively evaluating the high-frequency power (10–100 Hz) of LFPs using wavelet analyses (Figure 2). Most of the multi-unit activity (MUA) was concomitant with detecting the up state. Although the delta oscillations of at about 2 Hz were contaminated, the strong WPS of the slow oscillation up states was detected separately from the weak WPS of the delta oscillation. Therefore, we detected the up state containing a stronger high-frequency power than that for the down state. We compared control mice (Figure 2A) and PV-GAD67 mice (Figure 2B) in terms of state duration. PV-GAD67 mice displayed a shorter up state and a longer down state compared with control mice. Next, we compared control mice (Figure 2A) and SOM-GAD67 mice (Figure 2C) in terms of state duration. SOM-GAD67 mice displayed a longer down state compared with control mice. Our statistical analysis revealed that the up state of PV-GAD67 mice, but not SOM-GAD67 mice, was shorter in duration than that of control mice (Welch's two-sample *t*-test; *p* = 2.3 × 10^−3^; *N* = 5; Figure 3A). The down state tended to be of longer duration in PV-GAD67 and SOM-GAD67 mice than in control mice, although not significantly (Welch's two-sample *t*-test, Figures 2, 3B). Although the variability in down state duration appeared to be larger in SOM-GAD67 than in PV-GAD67 mice, no statistical difference was observed between them (Welch's two-sample *t*-test; *N* = 5, 4; Figure 3B). To investigate the relationship between MUA and up state duration, we also compared firing rate of MUA during the up state in PV-GAD67 and SOM-GAD67 mice with that in control mice (Figure 3C). The high firing rates with GABA antagonists contributed to shorter up state (Sanchez-Vives et al., 2010). We found that firing rate tended to increase in PV-GAD67 mice, whereas it tended to decrease in SOM-GAD67 mice. However, these differences were not significant (Welch's two-sample *t*-test; *N* = 5, 4; Figure 3C).

Therefore, the up state, but not the down state, was shortened specifically in PV-GAD67 mice. MUA during the up state tended to change asymmetrically between PV-GAD67 and SOM-GAD67 mice.

### Asymmetric alternation of delta oscillation in SOM-GAD67 and PV-GAD67 mice

The delta oscillations (1–4 Hz) in Figure 2, co-occurring with slow oscillations, appeared relatively stronger in SOM-GAD67 than in PV-GAD67 and control mice. To investigate the effects of inhibitory subtypes on delta oscillations, we compared the power of delta oscillations across mice strains (Figure 3D). Delta oscillation power tended to increase in SOM-GAD67 mice, whereas it tended to decrease in PV-GAD67 mice, although these tendencies were not significant (Welch's two-sample *t*-test; *N* = 5, 4). However, delta oscillation power tended to asymmetrically alternate between PV-GAD67 and SOM-GAD67 mice.

### CSD analysis of LFPs during the up state

**Figure 4:**
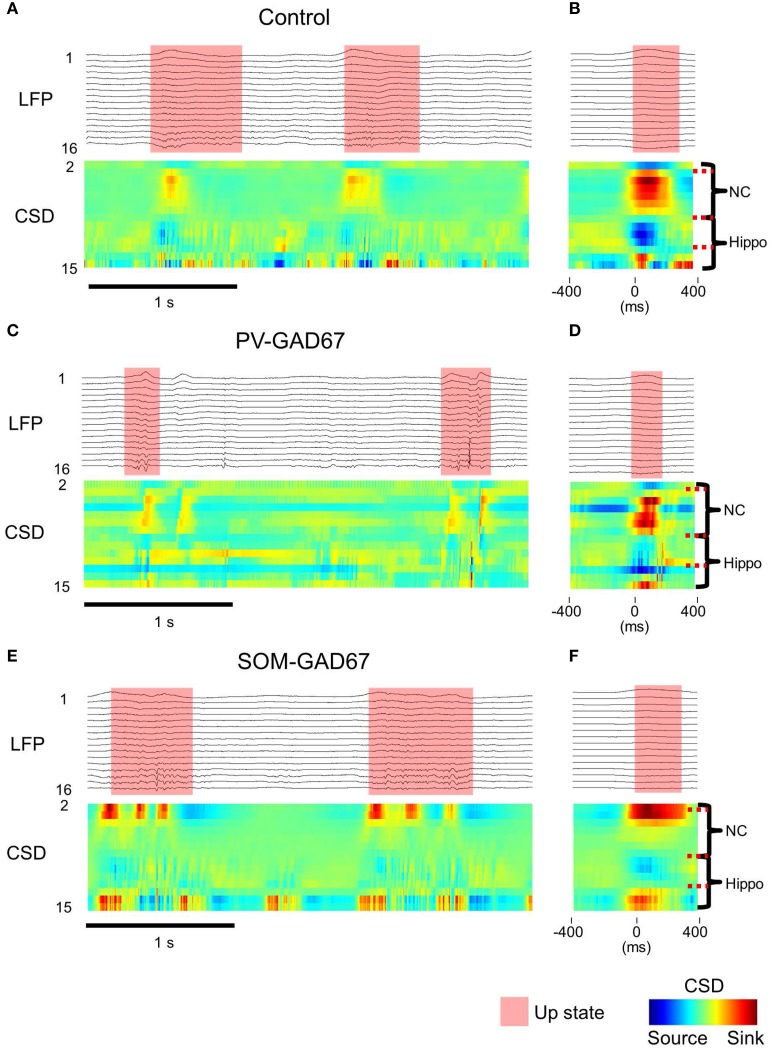
**CSD depth and temporal profile in the up state. (A)** LFPs example from all channels (upper) and the CSDs example calculated from the LFPs in control mice. **(B)** Up state start-triggered average example of LFPs (upper) and that of CSDs (lower) in control mice. The probe position according to the CSDs is placed on right side of the CSDs. The same calculations as described in **(A)** and **(B)** for the **(C,D)** PV-GAD67 mouse and for the **(E,F)** SOM-GAD67 mouse (semi-transparent red, up state; NC, neocortex; hippo, area around the hippocampus; colored bar, normalized CSD pattern).

We conducted a CSD analysis before investigating the origin of LFP oscillations with wavelet analysis and detected the sink and source patterns across the cortical layers. A CSD analysis removes volume conduction (i.e., leakage of electrical fields from an electrical primary current source through biological tissue toward measurement sensors). The sink is the inward current, which is mainly caused by excitatory postsynaptic potentials, and the source is the outward current, which is mainly caused passively by the sink (Mitzdorf, 1985). Our CSD profile examples in the control, PV-GAD67, and SOM-GAD67 mice are shown in Figures 4A,C,E. To eliminate noise, we averaged the CSD profile triggered at the start of the up state (Figures 4B,D,F). We found a sink in the neocortical deep layers and a source in the upper layers in control and PV-GAD67 mice. We found a sink in CA1 and a source in the dentate gyrus of the hippocampus. We also found a weak sink in neocortical deep layers although a strong sink in neocortical upper layers in SOM-GAD67 mice. These results are consistent with other studies. The neocortical CSD in the up state becomes the source in the upper layers and the sink in deep layers (Chauvette et al., 2010). In contrast, the hippocampal CSD of the up state becomes the source in areas around the CA1 and the sink around the dentate gyrus (Wolansky et al., 2006; Sharma et al., 2010).

### High frequency oscillatory depth profiles of the up state in interneuron subtype GAD67 knockout mice

**Figure 5:**
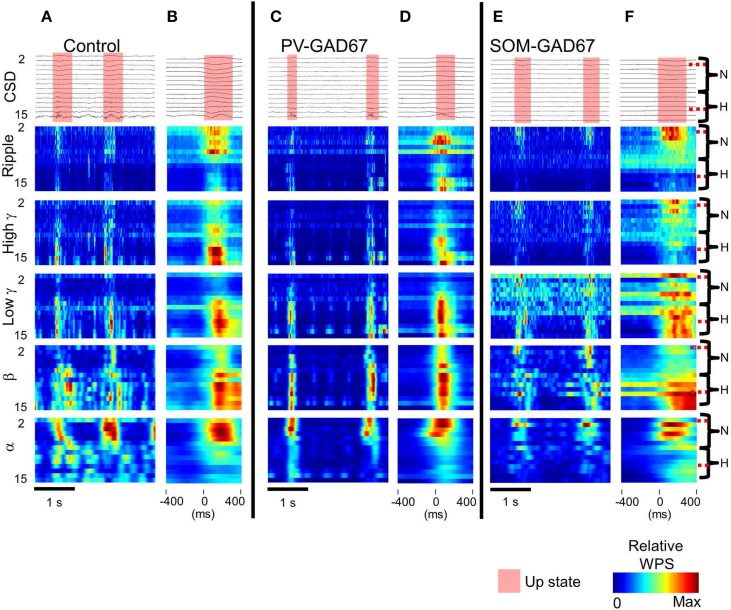
**The CSD WPS depth profiles in each high frequency band. (A)** CSDs (top) and WPS example calculated from the CSDs sorted according to each high frequency band (from the bottom alpha, 7–14 Hz; beta, 15–30 Hz; low gamma, 30–60 Hz; high gamma, 60–90 Hz; ripple, 100–200 Hz) in control mice. **(B)** Up state start-triggered average of CSDs (upper) and of WPSs (lower five rows) in control mice. N, neocortex; H, region around the hippocampus; semi-transparent red, up state; color bar, relative WPS. **(C)** The same as A, but for PV-GAD67 mice. **(D)** The same as **(B)**, but for PV-GAD67 mice. **(E)** The same as A, but for SOM-GAD67 mice. **(F)** The same as **(B)**, but for SOM-GAD67 mice.

**Figure 6:**
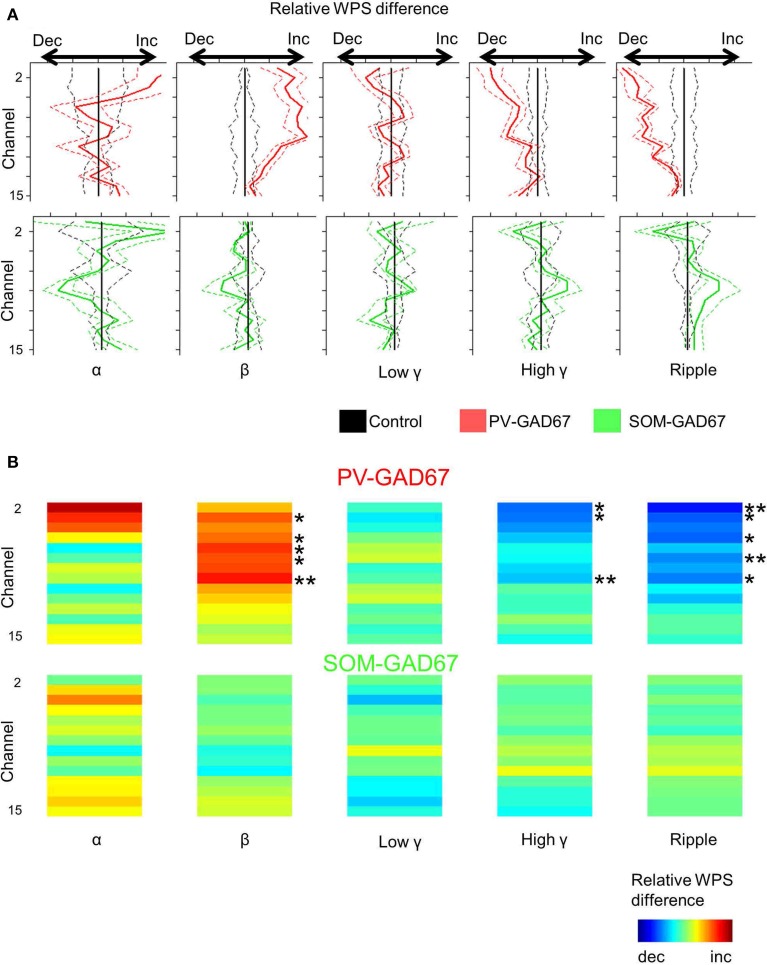
**Comparison of the CSD wavelet power spectrum depth profile in each high frequency band between PV-GAD67 or SOM-GAD67 and control mice**. **(A)** Comparison of the CSD WPS depth profile average in each high frequency band. Comparisons between the PV-GAD67 and control mice and between the SOM-GAD67 and control mice are shown in the upper and lower panels, respectively. The frequency range in each band from left (alpha) to right (ripple) is the same as that described in Figure 5. Red line, relative WPS difference between PV-GAD67 and control mice; green line, the difference between SOM-GAD67 and control mice; central line, the relative WPS of the control mice was set at zero. **(B)** Comparison of CSD WPS depth profile average among high-frequency bands as **(A)**, and its statistical results. Welch's two-sample *t*-test together with Benjamini-Hochberg correction, *N* = 5 PV-GAD67 mice, *N* = 4 SOM-GAD67 mice, *N* = 9 control mice; ^*^P < 0.05, **P < 0.01; error bars indicate SEM.

To investigate the depth profile of high frequencies across the cortical layers, we performed time-frequency analysis (wavelet analysis) of the CSD depth profiles (Lakatos et al., 2005; Maier et al., 2011). We classified the WPS into five frequency bands: alpha (7–14 Hz), beta (15–30 Hz), low gamma (30–60 Hz), high gamma (60–90 Hz), and ripple (100–200 Hz) bands. Here, we show a depth profile example consisting of five high-frequency bands in control, PV-GAD67, and SOM-GAD67 mice (Figures 5A,C,E). We also averaged the WPS aligned with the onset of the up state in the control, PV-GAD67, and SOM-GAD67 mice examples (Figures 5B,D,F). The PV-GAD67 and SOM-GAD67 mice examples exhibited many differences across layers compared with control mice example. For example, decreases in gamma and ripple in neocortical upper layers and increases in beta and low gamma in neocortical deep layers were observed in PV-GAD67 mice example (Figures 5B,D). Ripple and high gamma increased in the cortical upper layers, whereas low gamma increased in the neocortex, and beta decreased in the cortical deep layers of SOM-GAD67 mice example. We then calculated the difference in WPS between control and PV-GAD67 or SOM-GAD67 mice (Figures 6A,B). This population analysis revealed that part of these differences in WPS was reproduced. The high gamma and ripple power decreased in PV-GAD67 mice compared with control mice, particularly in the upper neocortical layers. Beta power also increased in PV-GAD67 mice, particularly in the deep neocortical layers. These changes were also shown in the examples (Figures 5B,D). In contrast, SOM-GAD67 mice showed little change compared with control mice. Little change was observed between the PV-GAD67 mice or SOM-GAD67 mice and control mice in the area around the hippocampus. Next, we tested these changes statistically (Figure 6B). The statistical analysis revealed that the high gamma and ripple powers decreased significantly in the neocortex of the PV-GAD67 mice. In addition, beta power in the neocortex increased significantly in PV-GAD67 mice (Welch's two sample *t*-test; *N* = 5; high γ, ripple, and beta; Figure 6B, upper). In contrast, no significant differences were observed between SOM-GAD67 and control mice (Welch's two-sample *t*-test; *N* - 4; Figure 6B, lower). Therefore, the gamma and ripple power decreased, particularly in the upper layers, and beta power increased, particularly in the deep layers in PV-GAD67 mice, whereas few changes were observed in SOM-GAD67 mice.

## Discussion

Our results demonstrate that up state duration of the slow oscillations was shortened in PV-GAD67 mice, but not in SOM-GAD67 mice. Furthermore, high gamma (60–90 Hz) and ripple (100–200 Hz) oscillation power decreased in the shortened up state, particularly in the neocortical upper layers, whereas beta oscillation (15–30 Hz) power increased in the neocortical deep layers. Delta oscillation power tended to increase in SOM-GAD67 mice, whereas it tended to decrease in PV-GAD67 mice. These results strongly suggest that PV cells specifically maintain the up state, high gamma and ripple oscillations in the upper layers and suppress beta oscillations in the deep layers. They also suggest that SOM and PV cells regulated delta oscillations.

A shortened up state in PV-GAD67 mice and a tendency for an increase in firing rate during the up state are consistent with previous studies using GABA antagonists. A GABA(A) antagonist induced a parametric shortening of up state duration in a dose-dependent manner. Cellular activities during the shortened up state, such as the firing rate, also increases (Mann et al., 2009; Sanchez-Vives et al., 2010). However, the involvement of GABAergic interneuronal subtypes in shortening of the upstate has not been shown. An important question is how PV-GAD67 mice, but not SOM-GAD67 mice, shorten their up state. Functional and anatomical differences exist between PV and SOM cells. PV cells inhibit the cell body and proximal dendrites of pyramidal cells, whereas SOM cells inhibit the distal dendrites (Martinotti, 1889; Fairèn et al., 1984; Kawaguchi, 1993; Kawaguchi and Kubota, 1993; Condé et al., 1994; Hof et al., 1999; Tamamaki et al., 2003). It is important to generate horizontal propagation within pyramidal cells in layer 5 during the up state, which may be propagation among cell bodies of layer 5 pyramidal cells (Markram, 1997; Wester and Contreras, 2012; Beltramo et al., 2013). Therefore, inhibiting cell bodies by PV cells could be the main inhibitory system, whereas inhibiting distal dendrites by SOM cells could be a sub-inhibitory system. Decreases in PV cells activity disinhibit cell bodies of pyramidal cells in the absence of GAD67, and their hyperactivity activates Ca^2+^- or Na^+^-dependent K^+^ channels (i.e., activity-dependent K^+^ channels) earlier, compared with control mice (Bazhenov et al., 2002; Compte et al., 2003; Hill and Tononi, 2005; Holcman and Tsodyks, 2006; Sanchez-Vives et al., 2010). Actually, our finding that MUA tended to increase in PV-GAD67 mice with a shortened up state was consistent with this interpretation. In contrast, SOM cell vacancy may have little effect on maintenance of the up state, during which distal input is not important. The CSD analysis also showed that the sink was concentrated in the deep layers during the up state, but not in the upper layers where distal dendrites gather. The sink is an inward current mainly caused by excitatory postsynaptic potentials (Mitzdorf, 1985). Therefore, our data strongly suggest that PV cells specifically contribute to persistence of the cortical up state.

The depth profiles of the high frequencies in PV-GAD67 mice were altered during up state of the slow oscillations; gamma and ripple oscillation power decreased in the neocortical upper layers, and beta oscillation power increased in the neocortical deep layers. We first considered how gamma and ripple power decreased in the upper layers of PV-GAD67 mice. The involvement of PV cells in gamma oscillations has been shown in several studies (Freund, 2003; Fuchs et al., 2007; Sohal et al., 2009; Buzsáki and Wang, 2012). Gamma oscillations are localized in the neocortical upper layer, whereas beta oscillations are localized in the neocortical deep layers (Roopun et al., 2006; Quilichini et al., 2010; Buffalo et al., 2011). The ripple oscillations observed in the neocortical up state are caused by fast-spiking interneurons, suggesting that gamma and ripple oscillations have similar mechanisms in the neocortex (Grenier et al., 2001). Although we treated high gamma and ripple oscillations separately, they could both be classified as high gamma oscillations. The decrease in gamma oscillations in the upper layers, as shown in PV-GAD67 mice, is consistent with these observations. Furthermore, patients with schizophrenia have reduced gamma oscillations, as well as reduced GAD67 expression (Gallinat et al., 2004; Akbarian and Huang, 2006). Indeed, a model study suggested that a reduction in the number of GAD67-expressing PV cells in patients with schizophrenia also reduces gamma oscillations (Volman et al., 2011). The PV-GAD67 mice used in the current study showed loss of GAD67 in PV cells. Therefore, these results suggest that the decrease in gamma oscillations in patients with schizophrenia are related to loss of GAD67 in PV cells *in vivo*.

We next considered how deep beta power increased in PV-GAD67 mice. According to a previous study, beta oscillations result from deep pyramidal cells connected via gap junctions, and they are enhanced by disinhibiting GABA(A) receptors (Roopun et al., 2006). Disturbing PV cells in the deep layers may enhance synchronization in the beta band by disinhibiting deep pyramidal cells. It has also been hypothesized that the triad of PV neurons, SOM neurons, and pyramidal cells is involved in beta oscillations (Vierling-Claassen et al., 2010; Li et al., 2013). Based on this hypothesis, disturbing PV cells may have enhanced beta oscillations by disinhibiting SOM cells. Our results suggest that PV cells are involved in balancing gamma and beta oscillations across layers *in vivo*.

We also found that delta oscillation power tended to increase in SOM-GAD67 mice, but tended to decrease in PV-GAD67 mice. Delta oscillations grouped as slow oscillations in the neocortex are assumed to be generated at two sites: neocortical delta and thalamic delta oscillations (Amzica and Steriade, 1998). However, inhibitory cell subtype involvement in delta oscillations has not been investigated. Furthermore, our data were limited to the neocortex. The relationships between delta oscillations and inhibitory cell subtypes will be studied in the future.

Our results showed a shortened up state, suggesting that PV cells maintain the up state and balance high-frequency oscillations across cortical layers. Based on the current findings and previous observations, we hypothesize that a series of events occurs from initiation to termination of the up state. Two possible mechanisms initiate the up state. The first is that spontaneous release of neurotransmitter activates pyramidal cells in the deep layers (Chauvette et al., 2010). The deep pyramidal cells play essential roles initiating and maintaining the up state (Sanchez-Vives and McCormick, 2000; Wester and Contreras, 2012; Beltramo et al., 2013). The second possibility is that the thalamus activates neocortical PV cells through spindle waves and initiates the up state. A group of fast-spiking interneurons fire early during the up state and phase lock to spindle waves from the thalamus (Puig et al., 2008; Ushimaru et al., 2012). Subsequently, PV cells in the upper layers are recruited to the up state by deep pyramidal cells or the thalamus, which initiate gamma oscillations and maintain the up state. Next, deep pyramidal cells maintain their activity via balanced suppression by PV cells. At the same time, gamma oscillations in the upper layers decrease gradually and beta oscillations increase reciprocally in the deep layers (Whittington et al., 1997; Faulkner et al., 1999; Olufsen et al., 2003). Finally, deep activity-dependent K^+^ channels are activated gradually, subsequently terminating cellular activity (Sanchez-Vives and McCormick, 2000; Bazhenov et al., 2002; Compte et al., 2003; Hill and Tononi, 2005; Holcman and Tsodyks, 2006; Sanchez-Vives et al., 2010). Our previous study also demonstrated that optogenetically stimulating the deep layers terminates the up state (Kuki et al., 2013). Therefore, PV cells not only balance high-frequency oscillations along the cortical layers, but they may also moderate cellular activity in the deep layers to maintain the up state.

Our results show that PV cells are involved in gamma–beta balance and up state maintenance. A question arises whether gamma–beta balance and up state maintenance are related. To answer this question, we conducted a statistical analysis of the relationship between beta–gamma balance and up state duration within each mice strain (data not shown). However, we found no significant correlations between them. Our findings suggest that high-frequency activity and slow oscillations are controlled independently.

Due to the limitations of our current study, we must consider long-term compensation in the knockout mice. We also have to consider the degree of contamination of PV cells undergoing Cre- mediated recombination in SOM-GAD67 mice. In SOM-IRES-Cre line, which was used for the generation of SOM-GAD67 mice in this study, 6–10% of neurons expressing a Cre-dependent reporter in any neocortical layers are fast-spiking/PV cells (Hu et al., 2013). For example, the SOM-GAD67 mice showed moderate changes in slow oscillations compared with control mice. We did not find any changes in the hippocampus between knockout and control mice, whereas previous studies revealed that SOM and PV cells play roles during slow oscillations (Sun et al., 2002; Fanselow et al., 2008; Buzsáki and Wang, 2012). Loss of GABA(A) receptors in the cerebellum has little effect on normal neuronal behavior due to homeostatic plasticity (Brickley et al., 2001).

In conclusion, our results demonstrate that PV cells play particularly important roles maintaining the up state and balancing the gamma and beta bands across the cortical layers. SOM and PV cells may make asymmetric contributions to the regulation of up-state duration, firing rate during the up state, and regulating delta oscillations. This study was performed on anesthetized mice. It would be interesting to extend the study to behaving mice to understand how PV cells are related to cognitive function. In particular, the shortage of persistent cortical activity in PV-GAD67 mice may have been related to the insufficient cognition in schizophrenia. To overcome the problem of compensation and to understand the oscillatory mechanism more precisely, it would be interesting to manipulate PV and SOM cell activities in each layer selectively and transiently using optogenetics.

## Authors and contributors

Toshinobu Kuki, Hajime Mushiake, and Yuchio Yanagawa designed research. Toshinobu Kuki, Kazuyuki Fujihara, Hideki Miwa, and Nobuaki Tamamaki acquired data. Toshinobu Kuki analyzed data. Toshinobu Kuki and Hajime Mushiake interpreted data. Toshinobu Kuki drafted the paper. Hajime Mushiake, Yuchio Yanagawa, and Hideki Miwa revised the paper. Hajime Mushiake finally approved the version to be published. Hajime Mushiake agreed to be accountable for all aspects of the work.

### Conflict of interest statement

The authors declare that the research was conducted in the absence of any commercial or financial relationships that could be construed as a potential conflict of interest.
